# An Atypical Presentation of Formic Acid Poisoning

**DOI:** 10.7759/cureus.7988

**Published:** 2020-05-06

**Authors:** Vishruth Vyata, Satya Durugu, Sahas Reddy Jitta, Shriya Khurana, Jaswanth Rao Jasti

**Affiliations:** 1 Internal Medicine, Osmania Medical College, Hyderabad, IND; 2 Internal Medicine, University of Louisville, Louisville, USA; 3 Internal Medicine, Kasturba Medical College, Manipal, IND

**Keywords:** formic acid, poisoning, : acute kidney injury

## Abstract

Although formic acid (FA) poisoning is rare, it is usually fatal. Many FA poisoning cases commonly involve rubber plantation workers in which these workers ingest FA accidentally or with suicidal intentions. This is a case presentation of FA poisoning by a 73-year-old man. Additionally, the patient’s old age likely contributed to his severe prognosis.

## Introduction

Formic acid (FA) poisoning is usually fatal and is not unusual among rubber plantation workers in third-world countries as it is readily accessible commercially with very minimal restrictions on trade and supply. Many of these workers often consume FA accidentally or with suicidal intentions. The economies of these relevant regions are primarily agriculture-based and rubber is a major agricultural product [1]. FA is often diluted before its usage as a latex coagulant for rubber production. Ingestion of FA causes metabolic acidosis and hemolysis, thus posing a risk for acute kidney injury. These workers often mix FA with alcohol prior to consumption. Corrosive in nature, FA causes upper gastrointestinal bleeding and those who survive are at risk of a tracheoesophageal fistula as a late complication. Management of such cases is usually based on symptomatic treatment. 

## Case presentation

A 73-year-old man with an alleged history of poison consumption was brought to the hospital. Neither him nor his family members were aware of the poisoning details. A detailed history revealed he lived with his daughter, who owns a rubber plantation. At the time of examination (four hours post consumption), it was found that 15-20 ml of concentrated FA mixed with alcohol was consumed. Following consumption, the patient experienced immediate oral burning sensations and retrosternal chest pain. The patient also experienced four episodes of hematemesis, each around 50-100 ml volume and a single episode of melena.

On examination, his vitals were stable and the patient demonstrated epigastric pain. An oral cavity examination revealed intense mucosal erosion and bloody saliva. Other systemic examinations revealed no abnormalities. The patient’s airway patency was ensured and saturation was maintained above 97%.

The patient’s arterial blood gas analysis revealed a pH of 7.26, bicarbonate (HCO3) concentration of 14.0 mmol/L, and partial pressure of carbon dioxide (pCO2) level 32.3 mmHg. Endoscopy of the upper gastrointestinal tract was deferred to 48 hours later to avoid perforation. His serum electrolyte reports were potassium 6.5 mmol/L and sodium 135.0 mmol/L. Additionally, his blood report showed his hemoglobin containing: 16.9 g/dl, red blood cell (RBC): 5.29 x 106 cells/mcL, total white blood cell (WBC): 23,000 cells/mcL, absolute neutrophil count: 19570 cell/mcL, total platelets: 265,000/mcL and total reticulocyte count was 1.71%. The man’s renal function test revealed serum urea of 28 mg/dL and creatinine of 1.4 mg/dL. His serum lactate dehydrogenase (LDH) was 1041 IU/L and creatine phosphokinase (CPK) level 506.0 IU/L. Other investigations were normal, hence generating an overall scenario in which the patient had metabolic acidosis with hyperkalemia and leukocytosis.

The patient was kept nil per oral and monitored continuously. His urine output was measured and arterial blood gas analysis was done every fourth hour for the first 24 hours. Intravenous fluid of 5% dextrose and normal saline was given. Proton pump inhibitor infusion was also started together with high doses of folinic acid. Sodium bicarbonate was also administered intravenously to counter the metabolic acidosis and was titrated according to the arterial blood gas analysis. The patient’s pain was managed with injectable tramadol. Gastrointestinal and psychiatric consultation was given. 

His urine showed cola color suggestive of hemoglobinuria and his urine output was also decreasing and by the second day of admission, he was anuric. On the second day, his serum urea escalated to 130 mg/dl and serum creatinine increased to 4.1 mg/dL. The patient’s serum potassium level also dropped to 5.3 mg/dL. On day three, his serum urea was 134 mg/dL and serum creatinine was 4.8, serum potassium level was 5.1 mmol/dl and his total leucocyte count was 13,600 cells/cm3. He continued to be anuric, hence, hemodialysis was started via his left femoral vein.

The patient continued to be dialysis dependent and required multiple sessions of hemodialysis. Hemodialysis was later performed via his right internal jugular vein. He subsequently improved following hemodialysis. However, during the fourth week of admission, the patient had multiple episodes of hematemesis and melena which led to hemodynamic instability. He was stabilized with ionotropic support and blood transfusions. Upon stabilization, an emergency upper gastrointestinal (UGI) tract endoscopy revealed an ulcer over the pyloric antrum. Hemostasis was secured with hemoclips and adrenaline injections. Symptomatically, the patient improved; however, persistent leukocytosis was still observed. Following a negative culture report of urine and sputum samples, his blood culture was analyzed. Urine culture was also observed as the patient was catheterized initially when the sputum sample was analyzed to rule out hidden aspiration pneumonia [2]. His blood culture revealed coagulase-negative staphylococci with a deranged coagulation profile - bleeding time: 3 minutes and clotting time: 6 minutes. It was determined he had sepsis with disseminated intravascular coagulation (DIC). Antibiotics were started according to culture sensitivity and the patient improved symptomatically. His internal jugular vein (IJV) hemodialysis catheter was changed and reports turned out to be negative. By the sixth week, his urine output started improving and hemodialysis was stopped. However, the patient developed urinary retention. In response, a Foley catheter was installed and removed two days later. Upon optimizing his medication, the patient was discharged. He was advised for regular follow-ups. The patient was free of symptoms after six months.

## Discussion

Though the details of the poisoning were not entirely revealed, a demographic history of the patient’s residence at a rubber plantation provided the first clue. Also, retrosternal burning chest pain, hematemesis, hematuria, vomiting, abdominal pain (our patient’s epigastric tenderness) are the most common indication of FA poisoning [3]. Although pungent and corrosive, FA is usually consumed with alcohol, just as this patient had done [2]. There were no complaints of dyspnea and normal vesicular breath sounds were heard bilaterally in all respiratory auscultatory regions suggesting that there were no symptoms of aspiration pneumonia - another complication of FA poisoning.

The patient’s arterial blood gas analysis showed low pH, low bicarbonate values, and normal carbon dioxide values confirming metabolic acidosis due to FA poisoning. High serum LDH and high CPK enzyme values with normal coagulation confirm the lysis of red blood cells due to metabolic acidosis solely. Initial high serum potassium level was due to lysis of red blood cells releasing intracellular potassium. However, persistent hyperkalemia was due to acute kidney injury. 

Acute kidney injury was anticipated at the start as the patient had dark cola-colored urine on admission despite normal renal function test. Investigation wise, acute kidney injury was evident from the second day of admission as clinically the patient was oliguric and this was further supported by sudden high levels of serum urea and creatinine. Despite starting on diuretics initially, the patient was worsening from oliguria to anuria. Hemodialysis was then performed as it is the best possible management for persistent hyperkalemia and acute kidney injury. Acute kidney injury occurs as a result of toxic insult of hemoglobin towards the renal parenchyma. The presence of hemoglobin in urine was indicated by the dark cola color of the urine. UGI endoscopy was initially postponed at admission as a history of hematemesis and worsening retrosternal chest pain gave suspicions of possible erosion of his esophagus. An immediate endoscopy would pose an increased risk of perforation in such situations [4]. 

A proton pump inhibitor infusion was necessary to reduce gastric acid production and prevent formation of gastric ulcers. Despite stress and inflammation, steroids were not administered as they may have precipitated impending gut perforation [4,5]. Folinic acid hastens FA degradation, hence it was given as an additional treatment [6].

The patient had a slow recovery from his acute kidney injury. This may be due to several factors: his old age, a higher concentration of poison consumed, and underlying sepsis. Though the source of infection was not found by investigation, it could have been due to a urinary tract infection or iatrogenic via internal jugular hemodialysis catheter. 

Oral ingestion of 10 ml FA is usually fatal and 15 ml usually causes death before even reaching the hospital [7]. However, this patient, despite consuming 15-20 ml of FA and alcohol, was successfully treated. Meticulous supportive treatment was the main contributor to successful treatment. Thus, this shows that the amount of the poison consumed should not be considered as a prognostic factor especially when patient history is unreliable or the poison has been mixed with another substance.

The importance of follow-ups in preventing long term complications was conveyed to the family members as well [8]. They were also advised to keep any potentially harmful substances away from him given his history of depression. The follow-ups were made by three departments: Nephrology, Gastroenterology, and Psychiatry for a more holistic approach. His kidney recovery was monitored with an emphasis on the appearance of late complications such as esophageal strictures. Most importantly, the antidepressant dosage was optimized to prevent another suicide attempt or suicidal ideations.

## Conclusions

Amongst rubber plantation residents, it is not unusual to suspect FA poisoning. It is important to repeat the renal function test despite a normal report in order to best anticipate acute kidney injury. The volume of poison consumed is not a good prognostic indicator as the precise amount may not be elicited from patient history. The best principle of management is supportive treatment while constantly monitoring for complications. Septic shock may also be a complication due to prolonged hospitalization and iatrogenic procedures which could possibly yield increased body stress.
